# Blastomere size in the human 2-cell embryo predicts the division order that leads to imbalanced lineage contribution to the future body

**DOI:** 10.17912/micropub.biology.001181

**Published:** 2024-05-20

**Authors:** Simon Zernicki-Glover, Nicola Stanislawska, Ekta M. Patel, Yu Hua Kavanagh, Maciej Meglicki

**Affiliations:** 1 Pasadena, CA, USA, Polytechnic School; 2 Warsaw, Poland, Akademeia High School; 3 Division of Biology and Biological Engineering, California Institute of Technology, Pasadena, California, United States; 4 Department of Physiology, Development and Neuroscience, University of Cambridge, Cambridge, England, United Kingdom

## Abstract

Retrospective tracing of somatic mutations predicted that most cells in the human body could be traced back to a single cell of the 2-cell stage embryo. Accordingly, a recent prospective study of the developmental trajectory of blastomeres in human embryos confirmed that progeny of the first 2-cell stage blastomere to divide generates more epiblast cells (future body). How the 2-cell blastomeres differ is unknown. Here, we show that 2-cell stage blastomeres in human embryos are asymmetric; they differ in size and the bigger blastomere divides first to 4-cell stage. We propose that this asymmetry might originate differences in cell fate.

**Figure 1. Analysis of blastomere size and time of division in 2-cell human embryos:**
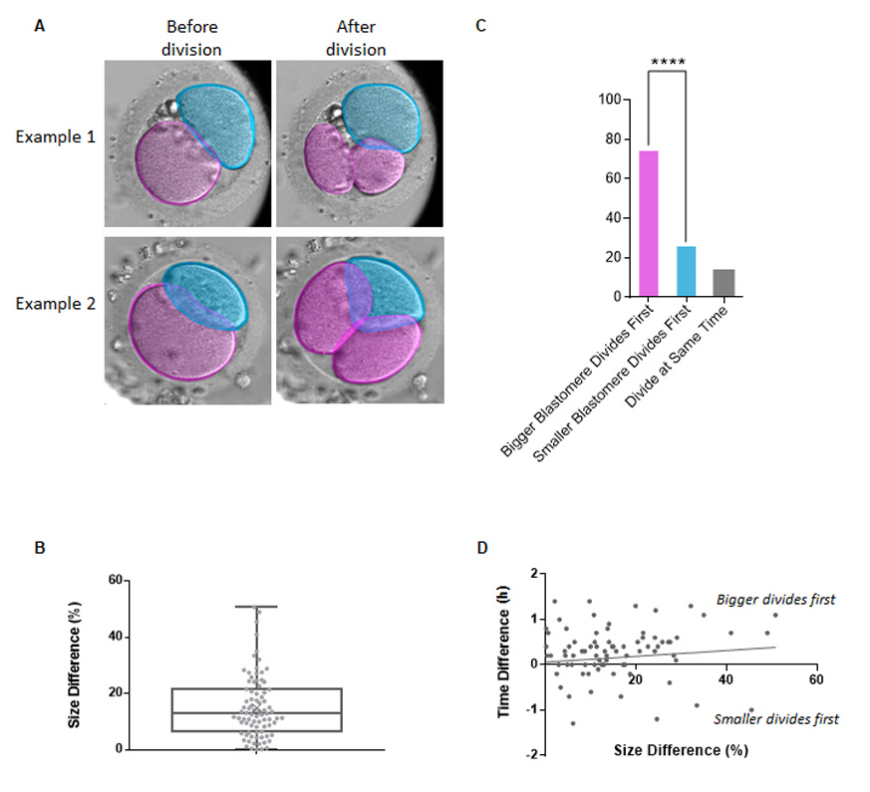
**A.** Schematic of the analysis used to generate data from human Embryoscope ^TM^ time-lapse movies. Two examples (single plane snapshots) from the movies are shown: before and after division of the bigger blastomere, pseudocolored in pink. The smaller blastomere is pseudocolored in blue. **B.** Quantification of the size difference between the two blastomeres of the 2-cell stage human embryo. Each datapoint represents the percentile difference between the two cells within individual 2-cell stage embryos. The centre line marks the median, the box contains the 25 ^th^ -75 ^th^ centile of the dataset, the whiskers mark the highest and lowest value, and the cross sign marks the mean. **C.** Quantification of order of division of blastomeres in 2-cell human embryos in relation to their size. Student’s t-test, p = 0.0000096 (****). n = 83 embryos. **D** . Scatter plot showing the correlation between % size difference (bigger/smaller) of the blastomeres of two-cell stage human embryos vs difference in time of their division (in hours, time of smaller blastomere division – time of bigger blastomere division). R = 0.04, p = 0.71.

## Description

Whether blastomeres of mammalian embryos show any tendency to follow a particular developmental pathway has been under debate for many years. During the first cleavage divisions of fertilized mouse eggs, blastomeres have similar morphology which is not indicative of their future fate. Cell fate specification becomes apparent in development when asymmetric cell divisions direct one daughter cell to the inside of the embryo and the other to the outside. Inside cells generate the inner cell mass (ICM) of the blastocyst, which gives rise to the pluripotent epiblast (the future body) and primitive endoderm/hypoblast (the future yolk sac), while the outer cells differentiate into extraembryonic trophectoderm (TE) (the future placenta) (Molè et al., 2020).

Developmental plasticity of mouse embryos led to a conclusion that all early blastomeres contribute equally to development, as removing an individual cell from the embryo (Tarkowski, 1959; Tarkowski et al., 2001, 2005, 2010) or cutting away their animal or vegetal poles is not detrimental for subsequent development (Zernicka-Goetz, 1998) . This contrasts to similar experiments in the deterministic, lineage-based development of many non-mammalian species, for example C.elegans (Munro & Bowerman, 2009) , Drosophila (St Johnston & Niisslein-Volhardt, 1992) and Xenopus (Houston, 2017) , where removing part of the embryo compromises specific aspects of development. Indeed both 2-cell stage, and most 4-cell stage, blastomeres of the mouse embryo contribute to both ICM and TE lineages. However, lineage tracing studies and gene expression analysis unexpectedly showed that 2- or 4-cell blastomeres have differing developmental potential, depending on their orientation and order of cell divisions (Bischoff et al., 2008; Casser et al., 2017; Gardner, 1997, 2001; Goolam et al., 2016; Krawczyk et al., 2021; Piotrowska et al., 2001; Piotrowska-Nitsche et al., 2005; Sun et al., 2020; Torres-Padilla et al., 2007; White et al., 2016) . Moreover, it was found that cells internalized at the 8- to 16-cell stage tended to contribute to the embryonic epiblast, whereas those internalized at the 16- to 32- or 32- to 64-cell stage tended to contribute to the extra-embryonic primitive endoderm (future yolk sac) (Morris et al., 2010) . The balance of inside and outside cells and so the composition of embryonic and extra-embryonic tissues is thus influenced by the number and timing of symmetric and asymmetric divisions in mouse embryos (Fleming, 1987; Johnson & Ziomek, 1981; Morris et al., 2010, 2013) . The unexpected nature of these findings made them contentious (Dietrich & Hiiragi, 2007) . However, support was gained from the discovery of molecular heterogeneities in gene expression and epigenetic modifications in mouse embryos that were shown to affect the contribution of a blastomere to specific lineages (Goolam et al., 2016; Lim et al., 2020; Panamarova et al., 2016; Torres-Padilla et al., 2007; J. Wang et al., 2018) .

In humans, the retrospective tracking of somatic mutations and lineage reconstruction recently predicted that in adults, the majority of human organs are derived from only one of two blastomeres at the 2-cell stage (Coorens et al., 2021; Fasching et al., 2021; Park et al., 2021; Spencer Chapman et al., 2021) . Similarly, retrospective tracking of mutations in the placenta and analysis of lineage contribution imbalances also led back to the 2-cell stage embryo (Coorens et al., 2021) . However, due to the retrospective nature of these studies, how these clonal differences arise remained unclear. To gain insight into this question, a recent study followed the developmental trajectory of blastomeres in live human embryos from the first cleavage division until the establishment of the three lineages in the blastocyst (Junyent et al., 2024) . This work showed that only one of the two 2-cell stage blastomeres contributes the majority of cells to the future body. Specifically, the first 2-cell blastomere to divide to the 4-cell stage has a higher likelihood of undertaking more asymmetric cell divisions, generating more of the epiblast, that will form the future body (Junyent at al., 2024) .

The above findings raise the question of whether, and if so how, the first two blastomeres differ to give this advantage in the timing of cell division. It was shown that in invertebrates asymmetric cell divisions in early development may play a role in establishing cell fate (Aoki & Shimizu, 2017; Munro & Bowerman, 2009; Ren & Weisblat, 2006; Shimizu et al., 1998; Wang & Seydoux, 2013) . Similarly, asymmetric cell divisions at the early stages of development are present in Xenopus laevis (Tassan et al., 2017) , which is an example of a vertebrate with deterministic type development. However, there are currently no well-documented examples of mammalian embryos displaying consistent blastomere size differences at the early cleavage stages. Although, in contrast to mouse, it has been observed that human blastomeres of the 2-cell stage embryo may differ in size, this observation has been linked to embryo quality, not the fate of the individual blastomeres (Hardarson et al., 2001; Holte et al., 2007) . We wished to test a hypothesis that differences in blastomere size at the 2-cell stage could be linked to their order of division. However, there are several limitations for studies using early human embryos. First, access to human embryos at the 2-cell stage is extremely limited. Research-consented embryos can be sourced from *in vitro* fertilization (IVF) clinics and this process relies on patient donation. However, early embryo freezing is no longer common clinical practice as embryos are commonly cultured to the blastocyst stage and only then would these be frozen for future use.

Thus, to approach this question, we acquired access to time-lapse movies of human embryo development obtained from an IVF clinic. The movies were acquired with the Embryoscope ^TM^ system and covered development from the zygote to the blastocyst stage. The movies used in this analysis were multi-focal time-lapse movies allowing us to trace blastomere division over time in several focal planes. Use of brightfield microscopy has limited potential for accurate segmentation and volumetric quantifications. Thus, as a proxy for overall blastomere size, we had to rely upon cell area at its mid-point, which approximates the maximal measured area between focal planes ( Fig. 1A, Methods ). We analysed movies of 83 dividing 2-cell stage embryos and measured the area of each blastomere 5 frames before division, recording which cell divided first ( Fig. 1A, Methods ).

We observed that overall, one blastomere had an area that was 15.3% (+/- 1.37%) larger than the other cell ( Fig. 1B ). Importantly, in 68.7% (57/83 embryos) of all embryos, the larger of the two blastomeres divided first ( Fig. 1C ). In the remaining 12.0% (10/83 embryos), the blastomeres divided at the same time and only in 19.3% (16/83 embryos) the smaller blastomere divided first. We did not observe a clear correlation between blastomere size difference and the degree of asynchrony ( Fig. 1D ).

The occurrence of unevenly sized blastomeres in early human development has been linked to potential aneuploidies and embryo quality (Hardarson et al., 2001; Holte et al., 2007) . However, our analysis included only movies of embryos that resulted in successful live births, which validates the quality of the embryos analysed and shows that differences in blastomere size in human embryos are not detrimental to embryo development.

The implication of these observations is that the first cleavage of the human zygote tends to divide the cell asymmetrically to generate daughter cells of differing size, which endows the larger cell with the capability of dividing ahead of its smaller sister cell at the next cleavage. In Junyent et al. (2024), it has been shown that the first blastomere to divide at the 2-cell stage generates daughter cells that also tend to divide first at the 4-cell and 8-cell stages, effectively giving it a higher probability to undergo the first asymmetric cell division and contribute preferentially to the ICM and so the future body. Although the quality of Embryoscope ^TM^ movies obtained from the clinic do not permit an accurate measure of cell volume, the result is nevertheless clear from these recordings. This finding also accords with earlier findings in the mouse embryo, where the 2-cell blastomere that divides first was found to contribute preferentially to the embryonic part of the blastocyst which contains the ICM (Piotrowska et al., 2001) , raising the possibility that this may be a common feature of mammalian embryos.

The mechanism behind the asymmetry of the first cleavage responsible for the difference in size between 2-cell blastomeres remains unknown. It was previously observed that the position and orientation of the first cleavage plane in the mouse embryo could be influenced by both the site of sperm entry into the egg (Piotrowska & Zernicka-Goetz, 2001; Plusa et al., 2002) and the site of the meiotic division in the oocyte (Plusa et al., 2002) , events which influence the organisation of the cortical cytoskeleton. In this case, it is possible that the zygote carries positional information established in the highly asymmetric meiotic divisions that influences subsequent cleavage events. However, a full mechanistic explanation of this phenomenon is still lacking. It cannot be discounted that the generation of 2-cell blastomeres of differing size is a stochastic event and that it is cell size per se that influences the timing of cleavage. It is plausible to imagine that the bigger blastomere may contain more organelles or other factors, such as mitochondria, which may result in a higher energetic status of this cell. Indeed, it has been shown that selective inheritance of distinct mitochondrial age-classes might act as a cell fate determinant in the asymmetric division of epithelial stem cell-like cells (Döhla et al., 2022). On the other hand, in the bigger blastomere some factors may be less concentrated in comparison to the smaller blastomere due to higher cytoplasmic volume. It has been shown human cell growth dilutes cell cycle inhibitor Retinoblastoma protein to trigger division (Zatulovskiy et al., 2020) . If similar inhibitors were present in the cytoplasm of 2-cell human embryos, they may be less concentrated in the bigger blastomeres. However, at these stage, the cells are not growing but beginning rounds of division into smaller cells at the onset of cleavage.

It is postulated that even small asymmetries between the blastomeres at the 2-cell stage can be amplified with time and translate to different cell fate (Chen et al., 2018) . This is consistent with the observations that asymmetries in the timing of division in 2-cell embryos could be linked to the asymmetries that contribute to the ICM during the 8- to 16-cell stage division (Junyent et al., 2024) . Our current observations point towards the influence of size differences between 2-cell blastomeres upon division timing. Future studies will be required to understand both the mechanisms whereby cell size exerts this effect, and the events that lead to the asymmetry of the first cleavage division of the zygote, resulting in this differential size between daughter cells.

## Methods

**Embryoscope ^TM^ time-lapse movies**

Embryoscope ^TM^ time-lapse movies capturing preimplantation development of human embryos from the zygote to blastocyst stage were provided by IVIRMA-Valencia (IVI Foundation, Spain). These movies were generated during routine IVF clinical practice, when the embryos were placed in time-lapse incubators. The use of movies for retrospective analysis was approved by the Research Ethics Committee of IVI Valencia (IRB protocol number 2203-VLC-028-MD). The identities of embryos that had been filmed were anonymized to the research team. Each movie contained transmitted light images on 11 focal planes with an average imaging frequency of 15 minutes. All samples represented embryos that resulted in successful pregnancies and live births, which validates the quality of the sample analysed.

**Measurements and quantification:**

Fiji (ImageJ) (Schindelin et al., 2012) was used to manually track blastomeres, annotate timing of the division and measure blastomere area. Movies with embryos positioned in a way that would make measurements inaccurate, embryos with blastomere morphology not allowing for accurate measurements, and low-quality movies were rejected from the analysis. Blastomeres were considered to have divided when the first frame with full completion of the cytokinesis could be observed. This timepoint was annotated as the timing of the division for each individual blastomere. Blastomere size measurements were taken 5 frames before the first sign of entry into mitosis could be observed (i.e. nuclear envelope breakdown and entry of nuclei into prophase, which can be clearly observed in bright field). The area of each 2-cell stage blastomere was used as a proxy for overall blastomere size. The focal plane with the largest blastomere area was always selected for the measurements.
